# Satisfaction with healthcare services among refugees in Zaatari camp in Jordan

**DOI:** 10.1186/s12913-021-06471-8

**Published:** 2021-05-26

**Authors:** Nesreen A. Salim, Shroq Hafedh Meyad, Faleh A. Sawair, Julian D. Satterthwaite, Samiha Sartawi

**Affiliations:** 1 Prosthodontic Department, School of Dentistry, The University of Jordan, Consultant in fixed and removable prosthodontics, Jordan University Hospital, Queen Rania Street, Amman, 11942 Jordan; 2 Bahcesehir University, Istanbul, Turkey; 3 Department of Oral and Maxillofacial Surgery, Oral Medicine and Periodontology, School of Dentistry, The University of Jordan, Jordan University Hospital, Amman, Jordan; 4 Restorative Dentistry, Division of Dentistry, School of Medical Sciences, University of Manchester, Oxford Road, Manchester, M13 9PL UK

**Keywords:** Syrian refugees, Healthcare services, Zaatari camp, Satisfaction, Dental services

## Abstract

**Background:**

Feedback on satisfaction regarding healthcare services is vital for continuous improvement of the service delivery process and outcome.

**Aims and methods:**

The objective of this study was to assess the satisfaction of refugees with the medical and dental services in Zaatari camp, under 3 domains with 20 key indicators (human and physical health resources, interaction and reactivity, and administration) using a self-administered questionnaire.

**Results:**

Of the 500 participants, the satisfaction rate was 72.5%. Young participants and participants with a shorter stay in the camp showed higher overall satisfaction rates (*P* ≤ 0.01). Within the domains, ‘interaction and reactivity’ achieved the highest satisfaction score, whereas ‘administration efficiency’ was ranked the lowest. As for elements within the domains, the most acceptable were the sufficient number of staff and the working hours, availability of radiological services and proper care for children, reasonable waiting time and asking for medical history in every visit. Whereas difficulty to access healthcare services, difficulty to be referred to hospitals, lack of follow up and lack of dental services were the least acceptable.

**Conclusion:**

In conclusion, whereas refugees were generally satisfied with the provided services, this study indicates that there are areas for further service improvement. This study highlights a significant gaps in healthcare services which if not addressed have the potential to amplify oral/medical health problems.

## Introduction

Refugees face serious hurdles; they must adjust to a new place and culture in unpredictable circumstances with an uncertain future, while at the same time attempting to cope with the tasks of everyday life [1]. Traumatic experiences in the country of origin, exacerbated by relocation, loss and chaotic and unhygienic living conditions in camps, can lead to extreme depression, panic attacks and crippling types of anxiety [2, 3]. Post-migration stress leads to poor mental health of refugees, greatly affects their emotional well-being, and sometimes results in a risk comparable to or greater than that of war-related trauma [4]. Stress is high during the resettlement period as refugees might be reminded of traumatic war experiences [2, 3]. Particularly, additional stress and systemic restrictions of refugee arrivals (such as socioeconomic conditions, poor accommodation, barriers to work, limited accessibility to healthcare, insecurity and in some cases, indefinite detention) may exacerbate existing stressors with long-term health inferences [5]. Reasons such as pre-migration trauma, fear of family security, near to death and forced separation from family members, and inferior levels of help-seeking actions lead to the aetiology and continuity of mental illness [3].

Health-related Quality of Life (HRQOL), including the physical, psychological and social aspects of health and well-being, is a significant indicator of the health impact of refugee experience. HRQOL data may serve a number of purposes, including identifying individuals and groups at high jeopardy for health issues, evaluating the distribution of health services, assessing community health needs, and assessing the efficacy of prevention and intervention plans and protocols [6].

Post-migration stress has a negative effect on (HRQOL) among newly arrived refugees and existing evidence indicates that this population has substantially higher oral disease levels than the general population [7].

Fast access to dental care may play a vital role in recovering from refugee experience, particularly for people who may have undergone torture resulting in trauma to the oral tissues (teeth, gums or mouth) [8].

The World Health Organization (WHO) has included patient satisfaction in the concept of quality of care assessment [9]. In that definition, it considers that: “Quality evaluation is an approach that ensures that every patient has a diagnostic and therapeutic approach that ensures the best clinical outcomes in accordance with current medical research, at the best cost, the best results in the least iatrogenic risk and greater satisfaction in terms of treatments, results and human interaction within the healthcare system” [10]. Patient satisfaction will reflect the efficacy of the healthcare system as it measures the perceived quality of the healthcare provided and must therefore be monitored constantly and frequently [11].

Due to the nature of medical services, the healthcare industry has a high degree of contact with the patient; researching the factors that affect patient satisfaction plays an important role in the growth of healthcare and in the performance of healthcare providers [11, 12]. The findings of such studies are progressively being used by healthcare professionals and regulators to identify and address issues and improve the quality of care [13]. Patient satisfaction may be measured on the basis of specific contributing factors, such as ease of access, waiting time, facilities and staff services, pre-testing of the transfer of information, physical and psychological distress induced by the examination, interpersonal skills, the humanitarian nature of the staff, the amount of information provided to the patient about his or her condition and the medical staff behaviours are the most important factors in defining patient satisfaction [11, 13].

The conflict in Syria has resulted in the displacement of over 5 million people [14]. Many of those have moved to a neighbouring countries, and 77,534 of them are now living in the Zaatari refugee camp in Jordan [14]. Refugees typically find it challenging to access dental services due to language and transport difficulties as well as cost, waiting lists and concerns about the quality of care provided [15]. The opinion of the users of healthcare services, as well as the evaluation of their satisfaction, are powerful tools that can lead to service improvement and, as a result, connect better the needs of users to the health services they are offered, guiding service provision and clinical management [12]. There is a lack of national refugee studies and no other known works in literature that have evaluated the quality of healthcare services provided to refugees in relation to the level of user satisfaction: there is thus a need to fill an information gap that could be useful for the improvement of the quality of services offered to this underprivileged population.

 The aim of this research was to investigate the level of patient satisfaction with the service quality provided in Zaatari refugee camp in Jordan and to determine the factors affecting service quality in the light of obtained data.

## Materials and methods

### Study group and design

The study was a cross-sectional study conducted at dental clinics at the Zaatari refugee camp between Sep 2019 and Nov 2019. All patients attending dental clinics in the camp as well as those accompanying them in the waiting areas. The target population was Syrian refugees ≥ 18 years old of both genders who live in Zaatari camp. Participants completed a structured questionnaire with closed questions (*n* = 500). The participation rate was 100 % (all participants who were invited consented to participate in the study). The sample size was calculated using the following formula [16]:

$$n = Z^{2} P (1-P) /d^{2}.$$

Where n = sample size, Z = 1.96 (level of confidence 95 %), *P* = 0.5 (expected proportion in population) and d = 0.05 (precision). According to this formula a sample size of about 400 is needed, in this study a sample of 500 was collected and analyzed.

### Data Collection, the questionnaire and validity

A self-administered modified questionnaire was used to assess patient satisfaction with the healthcare service provided in Zaatari camp: the questionnaire was in Arabic, the native language of the refugee population. The modified questionnaire used was a questionnaire based on a previous study [17]. In addition to the sociodemographic characteristics (age and gender), the final questionnaire included 20 questions/elements which were categorized into three domains: Human and physical health resources (9 elements), Interaction and reactivity (4 elements), and Administration (7 elements).

A Likert scale was used to collect responses with three options: satisfied (given a score of 3), neutral (score 2), and dissatisfied (score 1). The scores for each domain were calculated by adding the answers to all the elements in each domain and the total satisfaction score was calculated by adding the scores for the three domains. Therefore, the score range for the human and physical health resources domain will be (9 to 27), Interaction and reactivity (4 to 12), and Administration (7 to 21). The total satisfaction score will be in the range of (20 to 60). The satisfaction rate was measured by dividing mean domain score or mean overall score by the maximum possible score and then multiplying by 100.

Prior to the main study, the questionnaire was assessed for its validity by three health professionals who had experience in healthcare management. Additionally, pilot testing was undertaken with participants in the waiting area of the dental clinics assessing each question for clarity (*n* = 30). The initial questionnaire was modified following feedback from professionals and participants. A printed version of the final questionnaire was used to record participant answers.

### Statistical analysis

Statistical analysis was performed using SPSS for Windows release 16.0 (SPSS Inc., Chicago, IL, USA). Descriptive statistics were generated and Chi-square test, independent sample t-test, ANOVA test, Bonferroni Post Hoc test, and Spearman’s rank correlation test were used to examine associations between the different Sociodemographic factors. The significance level was set at *P* < 0.05.

### Results

The sociodemographic characteristics of the 500 study participants are shown in Table 1. Females outnumbered males and the majority were less than 40 years of age with more than 5 years of stay in the refugee camp, with none or primary school education.

**Table 1:** Sociodemographic characteristics of the studied sample

Variable		Number (%)
Gender	Male	223 (44.6)
	Female	277 (55.4)
Age	18–29	156 (31.2)
	30–39	176 (35.2)
	40–49	106 (21.2)
	≥ 50	62 (12.4)
Education	None/primary school	292 (58.4)
	High school	139 (27.8)
	College/university	69 (13.8)
Duration of stay in camp (years)	≤ 5	43 (8.6)
	6	123 (24.6)
	7	243 (48.6)
	≥ 8	91 (18.2)

Table 2 shows the median satisfaction score for each quality element, the satisfaction score for each domain, the total satisfaction score, and the associations of the various elements and scores with the sociodemographic variables.

**Table 2:** The median satisfaction score for each element, domain and overall satisfaction scores, and the associations of the various elements and scores with the sociodemographic variables

Quality elements		Median Satisfaction score	*P* value*			
			**Age**	**Gender**	**Education**	**Duration of stay**
**Human and physical health** **resources**	Ease of access to general & oral health facilities.	Dissatisfied	0.058	**0.015**	0.105	0.802
	Availability of all dental services.	Dissatisfied	0.193	0.831	0.558	0.772
	All family members are offered oral & dental examination and treatment.	Neutral	**0.049**	0.645	0.170	0.172
	The number of staff is sufficient to carry out all procedures in every visit.	Satisfied	0.272	**0.029**	0.553	0.111
	Proper care is available for children.	Satisfied	**0.009**	0.969	**0.001**	0.083
	Services for the elderly and those with special needs are available.	Neutral	0.057	0.952	0.322	0.579
	Prescribed medications are available in camp’s pharmacy.	Neutral	0.176	0.733	0.570	0.343
	Lab tests are available in the camp.	Neutral	0.549	0.596	0.968	0.162
	Radiological services are available.	Satisfied	0.245	0.752	0.251	0.734
	**Domain score**^**$**^ Mean ± SD	20.01 ± 3.23	**0.002**	0.524	**0.020**	0.881
**Interaction & reactivity**	Provide information about the available services in the medical center.	Neutral	**0.002**	0.984	0.966	**0.003**
	Provide oral health education to help understand, prevent and treat conditions.	Neutral	**0.004**	0.556	0.457	0.655
	Sympathetic attitude with patient’s problems.	Satisfied	0.388	0.825	0.612	0.347
	Staff communication skills.	Neutral	0.191	0.914	0.693	0.129
	**Domain score**^**$**^ Mean ± SD	8.98 ± 1.61	**< 0.001**	0.738	0.884	0.106
**Administration**	Announcements are made regarding the voluntary medical campaigns held in the camp.	Neutral	0.172	0.641	0.386	0.482
	Sufficient working hours.	Satisfied	0.138	0.359	0.172	0.628
	Waiting time before seeing my physician.	Satisfied	0.271	0.067	0.395	0.888
	Ask about medical and drug history in every visit.	Satisfied	0.418	0.080	0.254	0.933
	Contact patient if has a follow up visit and didn’t attend.	Dissatisfied	0.461	0.504	0.156	0.819
	Keep a medical file for each patient.	Neutral	0.408	**0.002**	0.181	0.476
	Ease of patient referral from clinic to hospital.	Dissatisfied	0.131	0.358	0.501	0.249
	**Domain score**^**$**^ Mean ± SD	14.51 ± 2.42	0.530	**0.034**	0.579	0.154
**Total**	**Overall satisfaction score**^**$**^ Mean ± SD	43.49 ± 5.85	**0.004**	0.584	0.130	0.260

*P value of Kruskal-Wallis test. ^$^*P *values for the scores was calculated using t-test or ANOVA test

### Human and physical health resources domain

The mean satisfaction score for the *human and physical health resources* domain was (20.01 ± 3.23) (satisfaction rate = 74.1 %). Significantly lower satisfaction rates were expressed by older patients (*P* = 0.002) and by those who had college or university education (*P* = 0.020). Females showed significantly lower satisfaction in ‘ease of access to health facilities’ (*P* = 0.015) but significantly higher satisfaction in ‘sufficiency of staff numbers’ (*P* = 0.029). Young patients were generally more satisfied regarding ‘involvement of all family members in examination and treatment’ (*P* = 0.049) and ‘delivery of proper care to children’ (*P* = 0.009). Participants with college/university education showed less satisfaction regarding delivery of proper care to children (*P* = 0.001).

### Interaction and reactivity domain

The mean satisfaction score for the *Interaction and reactivity* domain was (8.98 ± 1.61) (satisfaction rate = 74.8 %). Significantly lower satisfaction rates were expressed by older patients (*P* < 0.001).

Young patients were generally more satisfied regarding ‘availability of information about the available services in the medical centre’ (*P* = 0.002) and ‘oral health education’ (*P* = 0.004). Participants who had longer durations of stay at camp were less satisfied regarding ‘availability of information about the available services in the medical centre’ (*P* = 0.003).

### Administration domain

The mean satisfaction score for the *administration* domain was (14.51 ± 2.42) (satisfaction rate = 69.1 %). Significantly lower satisfaction rates were expressed by male participants (*P* = 0.034). Female participants were generally more satisfied regarding ‘availability of medical files for each patient’ (*P* = 0.002).

### Overall satisfaction

Participants in this study showed an *overall satisfaction score* of (43.49 ± 5.85) (satisfaction rate = 72.5 %). Significantly higher overall satisfaction rates were expressed by young participants (*P* = 0.004). The rank of the three domains is presented in Table 3.

**Table 3:** Overall ranking of domains related to satisfaction

Domain	Satisfaction rate%	Rank
*Interaction and reactivity*	74.8 %	1
*Human and physical health resources*	74.1 %	2
*Administration efficiency*	69.1 %	3
***Overall satisfaction score***	72.5 %	

## Discussion

Feedback on satisfaction regarding healthcare services is vital for continuous improvement of service delivery process and outcome [18]. Many studies have been conducted to evaluate patient satisfaction with healthcare services from different aspects using various measuring scales [13, 18–20]. However, no studies have evaluated refugee satisfaction regarding provided services. Limited availability/access to basic health services, including dental care, nutrition, immunization, preventive care and chronic disease management, is a major challenge to refugees [7, 15]. It is important to provide data to guide service provision and clinical management according to patient-based approaches. Furthermore, where gaps in data exist, it is important to identify key areas for research that can assist this process, such as evaluating patient satisfaction. The study population comprised a convenience sample from those who were attending dental clinics – as the outcomes of interest related to refugee opinion regarding dental and general health services, patients as well as those accompanying them were included as participants to allow results to be more generalizable (i.e. not restricted to the views of those receiving active care at that particular time); nonetheless the potential for bias remains with the possibility that those refugees who do not attend the dental clinics in any capacity having different views. Data from a broader and more representative sample from the refugee camp may give more confidence in the conclusions drawn, but exposure to the camp is strictly controlled and such access is not possible. In this research we evaluated refugee satisfaction regarding different aspects of oral healthcare services and it is found that the average percent mean score for satisfaction (20 elements) was 72.5 %.

Although the reported rate of satisfaction is considered moderate to high, it was still lower than that reported by Bedi et. al (89 %) who aimed to determine satisfaction with dental care services among the UK adult population using face-to-face home interviews of 5,385 UK residents and also lower than that reported by Mahrous M. (79.0 %) who conducted his study at the dental clinics of the College of Dentistry at Taibah University, Saudi Arabia using a self-administered (Arabic/English) questionnaire with a total of 202 patients. However, better than the satisfaction response reported by Othamn and Abdul Razak (61.7 %), as the objective of this study was to assess the satisfaction with school dental service provided via mobile dental squads in Selangor, Malaysia, under 4 domains of satisfaction: patient–personnel interaction, technical competency, administrative efficiency, and clinic setup using self-administered questionnaires by 607 participants [21–23]. Generally, this finding was similar to those reported by Othman and Jaafar, where more than 65 % of schoolchildren were satisfied with the care provided [24]. These comparisons should be made carefully because of the differences in the measuring scales, the tested domains and the populations under study.

Despite being lower than the studies cited above, the moderate to high satisfaction that has been reported in this study was higher than expected and may be explained by refugees being subjectively satisfied with their current situation compared to the transient pre-settlement unstable situation and the war-lived period which was deprived of any services before their life in camp [1]. It is worth noting that overall results on satisfaction do not highlight particular weaknesses of a service or problems encountered, and further investigations on specific aspect of care are required to disclose areas of expressed dissatisfaction [25].

The study covered three domains of satisfaction: *human and physical health resources*, *interaction and reactivity*, and *administrative efficien*cy. *Interaction and reactivity* were rated the highest, while *administrative efficiency* was rated the lowest. This study found that all elements of *Interaction and reactivity* were graded ‘acceptable’ to ‘satisfied’. However, ‘providing information about the available services’ and ‘providing information about the provided or needed treatment’ were less appropriate compared to the ‘sympathetic attitude of the service providers’ which was graded ‘very satisfied’. This finding is consistent with the previous research, where the most inappropriate trait found was that the dental operators did not clarify the technique prior to treatment [21]. Patients may feel that they have not been included in decision-making and that they have not been given an opportunity to be aware of the alternate treatments available to them and the risks associated with the procedures. Therefore, they will automatically fault the provider when care fails. [21]. On the other hand, about 46 % of participants in the previous study were disappointed with the operators’ personality, who were not happy, did not smile, and appeared grim (unattractive personality) and this is not in line with our findings. [21].

The present study found that most elements under *human and physical health resources* range from acceptable to very satisfied, except for ‘ease of access to general/oral health facilities’ and ‘availability of all dental services’. It has been reported previously that the attitude of non-attendance or delays in obtaining dental/medical treatment are strongly associated with accessibility, income, availability of healthcare facilities and perception of the need for routine dental care; all of which are major concerns for the refugee population [7, 26]. Furthermore, extended waiting times for emergency and routine dental treatment are associated with higher rates of amplified deterioration of teeth [27]. All of the factors listed above result in delay in pursuing dental care, and therefore leading to an extraordinary percentage of dental extractions [7, 26]. Consequently these two elements have negative and irreversible consequences on the health of this population, warranting an effective and targeted service with increased capacity. Additionally, modes of transportation were mainly on foot or by bicycle for nearby sectors, while for distances ranging from 4–5 km transportation by pickup trucks was shared for a fee, this factor again makes accessibility to certain clinics in different districts very difficult [7]. Regarding the available dental treatment, it has been reported that only basic restorative treatment and simple extractions are the most commonly available dental treatment in the camp [7, 26]. Expensive and sophisticated restorative, surgical, and prosthetic dental services are not available [7]. This can be explained by the lack of facilities and the need of costly specialist dental management, accompanied by lack of insurance or work-related benefits, being important barriers to accessing proper healthcare services [28].

This study showed that most of the elements under *administration efficiency* were rated acceptable to satisfied, except for ‘ease of patient referral from clinic to hospital’ and ‘contacting patient if has a follow up visit and didn’t attend’. This issue is highly important to be addressed as documentations and referral take months and sometimes years for specialized treatments in hospitals outside the camp. The camp only has small and few clinics that provide basic medical and dental services, lacking any specialized equipped hospitals for emergency or challenging cases [14]. No follow up for patients can be dangerous in medical fields, especially for certain life-threatening conditions that need regular follow up. There are many reasons why refugees may fail to attend health or dental appointments where they really need a regular follow up. These include conflicting resettlement priorities, administrative issues, language barriers, lack of transport, traditional health beliefs or misunderstandings, and previous negative health service experiences.[7, 15] This also reflects the lack of awareness and unfavorable attitudes toward the importance of maintaining teeth, oral health, and overall well-being and quality of life [7, 15]. This highlights the concept of ‘health literacy’: taking into consideration that most of the population in this camp are not educated (according to this and previous studies) they may lack the necessary decision-making skills to make value-based judgements regarding healthcare; moreover, the system itself (and administrative efficiency) may present barriers with this latter aspect perhaps having less influence as time in the camp increases; these issues may thus affect regular attendance and attitudes toward follow up appointments [29, 30].

This study reveals that there was a difference in perception between genders for some elements in some domains of satisfaction. This is in line with the study done by Othman and Jaafar who conducted a survey to test customer satisfaction with the school dental service among 16-year-old schoolchildren in the district of Tawau, Sabah, Malaysia and found that, generally, more female participants were dissatisfied with dental services [24]. In this study, for example, females showed significantly lower satisfaction in ‘ease of access to health facilities’. And this could be explained by living in a conservative society where females cannot walk and/or cannot go early morning alone, and this makes accessibility to services limited compared to males who can go anywhere, anytime in the camp independently.

On the other hand, males were more dissatisfied with the administration domain than females and this is in line with previously reported results [31]. Additionally, duration of stay in camp influenced the level of satisfaction. Where refugees with longer stay were less satisfied. This could be explained by being exposed to more previous negative health services experiences than refugees with shorter stay.

Age and education in this study have also shown an influence on the level of satisfaction. In contrast, a previous study on satisfaction with previous dental care in a Swedish population, found that age and gender did not influence the level of satisfaction [32]. Hall and Dornan’s meta-analysis concluded that sociodemographic characteristics such as gender are a minor predictor of satisfaction [31]. In this study, higher overall satisfaction rates were expressed by young participants. This result was in contrast to that reported previously where greater age showed greater satisfaction [31], but is in agreement with a study done on 400 participants as part of community survey in a mid-Atlantic state by Weiss [33]. Participants with higher education showed less satisfaction regarding certain services such as ‘delivery of proper care to children’. This finding is in agreement with a previous study that found that less education is associated with greater satisfaction [31]. This may be explained by the greater import given to health by individuals with higher socioeconomic status (education) [29]. Perhaps more educated patients apply higher standards in evaluating their care that even the objectively better care they receive does not seem as subjectively satisfying as the care rendered to less educated patients [31]. However, another study concluded that relations based on education were completely inconsistent [33], and highlighted that certain predispositional factors (confidence in the community medical care system, having a regular source of care, and being satisfied with life in general) are more important predictors of patient satisfaction than patient age, sex, race, educational attainment, or income [33].

## Conclusion

There is a lack of population-based data identifying refugee satisfaction regarding provided healthcare services. This study can be used as a guide for policy makers and humanitarian organizations to improve patient satisfaction as an indicator for the quality of health services and the perceived value of the services, being part of the total quality management policy of the hosting countries, and to implement targeted health promotion and preventive programs. All elements/indicators that were highlighted as negative factors in this paper by refugees can be resolved effectively when there is good understanding and collaboration between authorities and community organizations. For example, a potential solution to be considered regarding the issues of treatment cost limitations and availability of services and facilities to provide these treatments (especially beyond primary healthcare) is to arrange for and provide treatment in collaboration with dental/medical schools in major universities/hospitals in a mutually beneficial fashion, where students’ requirements for graduation can be more easily met by providing treatment to refugees. It is worthwhile to note that, based on this study, determinants of satisfaction are not only related to costly interventions and the development of complex medical/dental services, other individual factors such as communicative skills of the staff, listening to patient complaints and provision of detailed explanation of treatment, play crucial role on patient satisfaction.

Based on this study the following conclusions were drawn for the population group attending dental clinics:

1. Refugees were generally satisfied with the provided services with 72.5 % satisfaction rate.
2.  Young participants and participants with a shorter stay in the camp showed higher overall satisfaction rates.
3. ‘Interaction and reactivity’ domain achieved the highest satisfaction score, whereas ‘administration efficiency’ was ranked the lowest.
4. As for elements within the domains, the most acceptable were the sufficient number of staff and the working hours, availability of radiological services and proper care for children, reasonable waiting time and asking for medical history in every visit. Whereas difficulty to access healthcare services, difficulty to be referred to hospitals, lack of follow up and lack of dental services were the least acceptable.

## Data Availability

All collected data from patients analysed during this study are included in this published article. Some datasets are available from the corresponding author on reasonable request.

## Abbreviations

HRQOL: Health-related Quality of Life
WHO: World Health Organization
